# Insulin Resistance and Urolithiasis as a Challenge for a Dietitian

**DOI:** 10.3390/ijerph19127160

**Published:** 2022-06-10

**Authors:** Michalina Lubawy, Dorota Formanowicz

**Affiliations:** Chair and Department of Medical Chemistry and Laboratory Medicine, Poznan University of Medical Sciences, 60-806 Poznan, Poland; michalina.lubawy@student.ump.edu.pl

**Keywords:** insulin, insulin resistance, diet, dietitian, urolithiasis, inflammation

## Abstract

Many obesity and diet-related diseases have been observed in recent years. Insulin resistance (IR), a state of tissue resistance to insulin due to its impaired function, is a common coexisting condition. The most important predisposing factors are excessive visceral fat and chronic low-grade inflammatory response. However, IR’s pathogenesis is not fully understood. Hence, the diagnosis of IR should be carried out carefully because many different diagnostic paths do not always give equivalent results. An additional disease that is often associated with IR is urolithiasis. The common feature of these two conditions is metabolic acidosis and mild inflammation. A patient diagnosed with IR and urolithiasis is a big challenge for a dietitian. It is necessary to check a thorough dietary history, make an appropriate anthropometric measurement, plan a full-fledged diet, and carry out the correct nutritional treatment. It is also essential to conduct proper laboratory diagnostics to plan nutritional treatment, which is often a big challenge for dietitians. The diet’s basic assumptions are based on the appropriate selection of carbohydrates, healthy fats, and wholesome protein sources. It is also essential to properly compose meals, prepare them, and plan physical activities tailored to the abilities. The study aims to summarise the necessary information on IR with concomitant urolithiasis, which may be helpful in dietary practice.

## 1. Introduction

The advancement of technology, the increasing availability of caloric fast food, a decrease in society’s physical activity, and a sedentary lifestyle have led to a significant increase in society’s average weight [1]. The prevalence of obesity has increased worldwide over the past 50 years, reaching a pandemic level [2]. Along with civilisation’s progress, obesity-related diseases such as type 2 diabetes, hypertension, lipid disorders, and urolithiasis are increasing [3,4]. Tissue resistance to insulin is a common condition that coexists with these diseases. The pathogenesis of insulin resistance (IR) is not yet fully understood. The studies suggest that mutations, excessive visceral fat, chronic low-grade inflammation, and prolonged excessive sympathetic stimulation may be predisposing factors [5,6,7,8]. A properly selected and balanced diet may help increase tissue sensitivity to insulin, reduce the risk of many diseases, and achieve the desired body weight value [9]. Due to disturbed fat metabolism and abnormal carbohydrate metabolism found in IR, a patient with this disorder often challenges a dietitian. The association of IR with urolithiasis seems to be even greater. This study aimed to summarise the knowledge about IR and urolithiasis that can be useful in a dietitian’s practice.

## 2. Pancreas, Insulin, and Diabetes

One of the critical mechanisms for the proper functioning of the body is to maintain an appropriate level of glucose in the blood. The pancreas plays a significant role in the body’s carbohydrate metabolism by secreting insulin or glucagon. Abnormalities in the secretion of these hormones can lead to disturbances in the body’s homeostasis and, consequently, to disease states such as IR. When the blood glucose level decreases, the pancreas releases glucagon, which causes glycogenolysis in the liver and muscles, raising blood glucose levels [10]. Insulin, an anabolic peptide hormone secreted as a stimulus-response in a postprandial increase in blood glucose, plays the opposite function to glucagon. Its action at the cellular level involves the metabolism of amino acids, lipids, and carbohydrates. It facilitates the diffusion of glucose into fat cells and muscles by modulating glucose transport protein 4 (GLUT4) translocation. GLUT4 is the most crucial transporter for the IR glucose uptake into cells; it requires adequate insulin for proper function [11]. Insulin increases the synthesis of glycogen and reduces its breakdown. It stimulates the synthesis of fatty acids and TAG storage in adipose tissue and inhibits the oxidation of fats. It supports amino acid synthesis by influencing mRNA transcription and ribosomal mRNA translocation [11,12].

**Figure 1:**
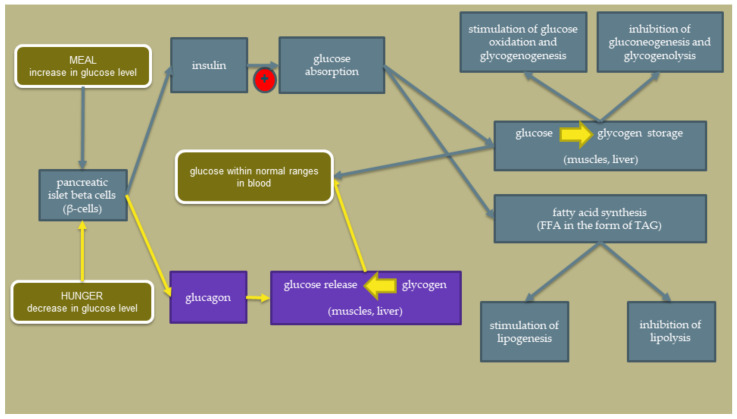
The insulin action is in a healthy state. (FFA—free fatty acids; TAG—triglycerides).

The scheme showing the insulin action in a healthy state is shown in Figure 1. The red circle points to the critical problem appearing in IR. The National Health and Nutrition Examination Survey (NHANES) determined that fasting circulating insulin levels in healthy subjects should range between 25 and 70 pmol/L [13]. After taking a meal, the insulin level may increase to about 300–800 pmol/L [14].

Insulin secretion is a highly regulated process in the human organism. Our understanding of the processes underlying effective-regulated insulin secretion is hampered by the complex structure of the pancreas that supports insulin secretion. Insulin release is stimulated by the growth hormone, cortisol, prolactin, and gonadal steroids, which are decreased by parathormone (PTH) action. The effects of thyroid hormones are more diverse. Epinephrine and sympathetic nerve stimulation inhibit insulin release. In turn, cholinergic stimulation promotes insulin release. Moreover, pro-inflammatory cytokines diminish the effect of insulin and have been implicated in the IR phenomenon [11,12].

The signal about the attachment of insulin is sent to the cell via insulin receptors (IR) after the insulin molecule attaches to the appropriate place. It forms a large signalling protein complex on the cell membrane surface around glycoproteins, which are the cytoplasmic domains of the IR. These domains are found mainly in metabolically active tissues and have two extracellular α and two intracellular β subunits. The binding of insulin to IR causes insulin receptor dimerisation and autophosphorylation of their tyrosine residues inside the cell, allowing the binding of the insulin receptor substrate (IRS) proteins [15]. There are two types of IRS protein in humans—IRS1 and IRS2. Both can be substrates for IR but have different functions. Insulin action starts after insulin binding with its receptor on the cell’s surface, activating at least three signalling pathways: (1) via phosphokinases: phosphoinositide 3-kinase (PI3K) and protein kinase B (PKB), which mediate insulin’s anabolic effect: glucose uptake, glycogen synthesis, de novo lipogenesis, and protein synthesis; (2) via adaptor protein (SHC) and mitogen-activated protein kinases (MAPKs): mitogen-activated protein kinase/extracellular signal-regulated kinase (MEK/ERK) pathway, which leads to cell proliferation and protein synthesis; (3) via NADPH oxidase 4 (NOX4), a dual phosphatase with both protein and lipid phosphatase activities, inhibiting phosphatase and tensin homolog deleted on chromosome ten (PTEN). PTEN-regulated metabolic functions act paradoxically toward insulin sensitivity [16].

According to the studies [15,17], hyperinsulinemia can be treated as a condition predicting type 2 diabetes. It has been confirmed that insulin malfunction precedes hyperglycemia development among people at increased risk of developing type 2 diabetes, and patients with type 2 diabetes have suffered from IR over the years. Among this group, hyperglycemia occurs when pancreatic β-cells, after prolonged production of large amounts of it, cannot produce enough insulin to compensate for its defect. Cells exposed to elevated insulin levels stop responding correctly to insulin signals constantly. This is mainly because of the impaired signal transduction due to receptor dysfunction. In response to a long-term increase in insulin levels, subsequent stages of the phosphatidylinositol 3-kinase (PI3K)/protein kinase B (AKT) signaling pathway are disturbed, which means that AKT does not stimulate translocation to the GLUT4 cell surface in myocytes and adipocytes. This results in a disturbance in glucose absorption, gluconeogenesis, and lipolysis with simultaneously properly occurring lipogenesis, which in turn causes the deposition of adipose tissue. Both too little and too much insulin are a threat to the body. Hyperinsulinemia causes diabetes associated with insulin deficiency and disrupts the physiological function of cells [15,17].

It has also been confirmed that hyperinsulinemia due to improper diet precedes the development of IR [15].

## 3. Insulin Resistance

**Figure 2:**
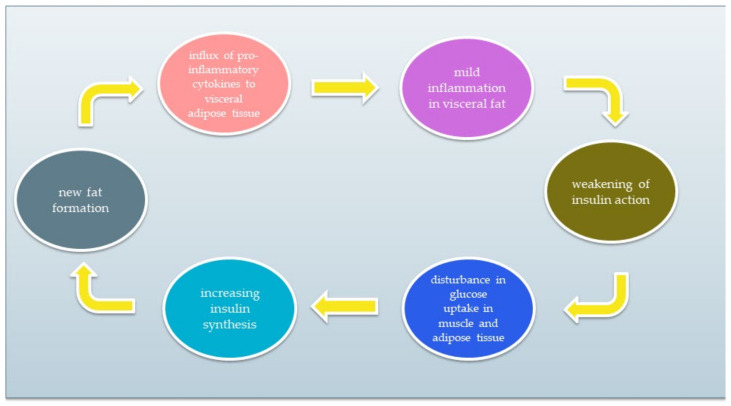
The vicious circle of insulin resistance.

IR is a condition of impaired insulin action by which glucose homeostasis is disturbed. As a result, tissue response to insulin is reduced while maintaining normal or elevated blood levels. Resistant tissues cannot properly metabolise glucose that remains in the blood. As a result, pancreatic β cells do not stop producing insulin. Excessive insulin synthesis leads to increased tissue resistance to insulin, forming a vicious circle. A simplified diagram of the vicious circle is shown in Figure 2. The chronic inflammatory response and low activity cause IR development [5,6,18,19,20]. The reasons for IR include (1) insufficient physical activity, (2) visceral fat level, (3) impact of some types of drugs, and (4) genetic factors. Moreover, it has been discovered that the development of IR can be associated with several conditions, such as cardiovascular diseases, non-alcoholic fatty liver disease, strokes, or polycystic ovary syndrome. IR significantly increases the risk of type 2 diabetes and metabolic syndrome [20,21,22,23].

There are two types of IR: hepatic and peripheral. Hepatic IR is characterised by increased glycogenolysis and gluconeogenesis in hepatocytes and increased very-low-density lipoprotein cholesterol (VLDL-C) and triglycerides synthesis. In turn, peripheral IR refers to skeletal muscle and fat. Then, glucose uptake by skeletal muscles is impaired, and fat distribution in adipose tissue increases, which leads to the release of free fatty acids (FFA) [24].

IR can also be divided into pre-receptor, receptor, and post-receptor disorders. The pre-receptor disease is characterised by abnormal insulin molecule structure. An example would be an endogenous insulin molecule’s genetically conditioned, abnormal structure. In this case, when the exogenous insulin is administered, glucose absorption into the cell is normal. Receptor IR occurs when a gene responsible for the insulin receptor’s structure or function is mutated. Mutations cause inappropriate binding of insulin to the receptor. Post-receptor IR occurs in the case of disorders of processes signalling the attachment of insulin to the insulin receptor or diseases of the structure and operation of glucose transporters to the cell [24].

**Figure 3:**
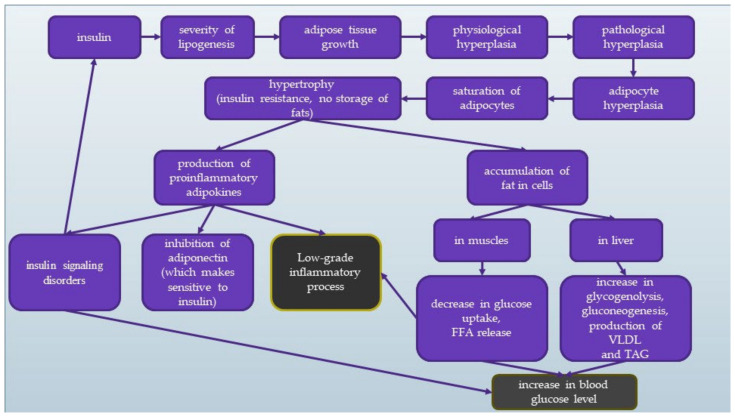
Mechanism of insulin resistance. (FFA—free fatty acids; TAG—triglycerides; VLDL—very-low-density lipoprotein).

The scheme of the mechanism of IR has been presented in Figure 3. Blue squares reflect the insulin changes in the body, and grey shows the final effect leading to increased blood glucose levels and low-grade inflammation.

## 4. Factors Affecting the Development of Insulin Resistance

### 4.1. Activating Factors for Pro-Inflammatory Pathways and Genetic Factors

Many years ago, IR began to be combined with inflammation in tissues. In 1901, Williamson published a study on the possibility of treating diabetes with high doses of salicylates [19,25]. Less than a hundred years later, Hotamisligil et al. showed that tumour necrosis factor (TNF-α) expression is increased in adipose tissue of obese mice, and its neutralisation causes an increase in tissue sensitivity to insulin [26,27,28].

In recent years, there has been much talk about the role of the chronic low-grade inflammatory response in the pathogenesis of IR. Inflammatory mediators, such as TNF-α, interleukin 1β (IL-1β), and interleukin 6 (IL-6) found in both adipose tissue and macrophages and other cells, may activate pro-inflammatory pathways and lead to low-grade inflammation [26,27,28]. Obese patients have been shown to have elevated pro-inflammatory cytokines and their receptors. Animal studies have shown that partial or complete silencing of genes encoding TNF-α or IL-1α leads to increased insulin sensitivity to peripheral tissues. Animals modified this way showed lower blood glucose and insulin levels than those whose gene expression was not altered [29,30]. A genetic factor affecting the development of IR may also be a mutation affecting the structure or function of insulin receptors on the cells’ surface. They allow for the proper attachment and action of the insulin molecule and thus maintain homeostasis in the body [31]. It is also worth noting that obesity and increased inflammation may contribute to an increased risk of atherosclerosis through epithelial damage and vasodilatation, and excess free fatty acids (FFA) [32]. Animal studies have shown that lowering inflammation in the body is usually associated with improved tissue sensitivity to insulin and its metabolic functions, so there is a chance that a therapy supporting IR based on this mechanism will be developed [33].

### 4.2. The Impact of Adipose Tissue on IR

The distribution of adipose tissue is of great importance in developing insulin resistance. Subcutaneous adipose tissue is more sensitive to the anti-lipolytic action of insulin and therefore releases less FFA. Visceral fat plays a more critical role in developing this condition.

Adipocyte hypertrophy causes an increase in the production of protein substances that increase IR [34]. Moreover, adipose tissue hypertrophy causes adipocyte hypoxia, which leads to increased cellular stress and activation of JNK (c-Jun N-terminal kinases) [35]. Another factor associated with IR in adipocyte hypertrophy is the type of macrophage found in adipose tissue. Macrophages, phagocytic cells activated by cytokines, participate in the body’s defence mechanisms by absorbing apoptotic bodies. There are two main types of macrophages, i.e., M1 (classically activated) and M2 (alternatively triggered by exposure to specific cytokines). The latter occurs in people with slim bodies. They take part in tissue repair processes and prevent the development of inflammation, thus inhibiting the growth of obesity. They also stimulate the synthesis of anti-inflammatory cytokines such as IL-4, IL-10, and IL-13 [36,37,38]. The development of obesity causes hypertrophy of adipocytes, secretes pro-inflammatory cytokines, and causes the influx of monocytes to adipose tissue. It results in the accumulation of M1 macrophages in adipose tissue. M1 macrophages are classic macrophages that occur mainly in obese people. They are responsible for the development of inflammation. When stimulated, they release TNF-α, IL-6, or IL-12, while inhibiting the synthesis of anti-inflammatory IL-10 [11,28,38].

Obesity causes an influx of macrophages to adipose tissue, proportional to the degree of obesity. There are distinct groups of macrophages around the adipocyte matrix in people with excessive adipose tissue—“Crown-like structures” (CLSs). Their presence may be associated with the weakening of phagocytosis [39].

### 4.3. Metabolic Syndrome

Scientific research indicates a relationship between the occurrence of IR and urolithiasis with diet and lifestyle [40,41]. Increasingly, attention is paid to the coexistence of this disease with the metabolic syndrome (MS) components, such as obesity, diabetes, hypertension, or lipid profile disorders [40,42,43]. The criteria for diagnosing metabolic syndrome are presented in Table 1 [44]. The characteristic IR FFAs are released from the excessive visceral adipose tissue that often characterises MS patients. An excess of FFA causes a decrease in tissue insulin sensitivity, an increase in glucose levels, and an abnormal lipid profile, leading to dyslipidemia, hypertension, type II diabetes, and inflammation [45]. Studies suggest that weight gain positively correlates with the possibility of developing urolithiasis, especially among women [46]. Research by Kohjimoto et al. [47] showed that patients with MS reveal a 1.8 times greater risk of recurrent stone formation than patients without these features. The presence of insulin resistance is not considered a diagnostic criterion. Still, scientific research indicates its association with both urolithiasis, and Spatoła et al. [48] suggest the necessity of screening for MS among people suffering from urolithiasis, suggesting the presence of stones may be closely related to IR.

## 5. Acid-Base Homeostasis

Acid-base homeostasis is essential for the proper state of the body. Maintaining an arterial blood pH between 7.36 and 7.44 is vital for maintaining normal health. When the pH is too low, it is called metabolic acidosis; when it is too high, it is called alkalosis [49]. The characteristic symptoms of metabolic acidosis are a decrease in the serum bicarbonate (HCO_3_^−^) concentration, a decrease in the arterial partial pressure of carbon dioxide (PaCO_2_) and a reduction in blood pH. We distinguish between the acute form, which lasts from a few minutes to several days, and the chronic form, which lasts several weeks to several years. The leading causes of chronic metabolic acidosis include the loss of bicarbonate and impaired acidification associated with renal dysfunction [50].

## 6. Urolithiasis

Urolithiasis is one of the oldest known diseases. Through archaeological research of Egyptian mummies, it has been proven that this disorder affected society in antiquity. It is characterised by insoluble deposits (stones) in the urinary tract formed due to the crystallisation of components present in the urine [51]. These stones often cause discomfort in the form of acute colic pain, nausea, and vomiting. They range in size from a few micrometres to a few centimetres. It is estimated that about 97% is in the kidneys and ureters, and 3% is in the bladder and urethra [52]. Thanks to technological advancement, we now know that stones can consist of about 100 different chemical compounds and 80% of them mainly contain calcium oxalate (CaOx). Besides calcium oxalates, we usually find calcium phosphate and gout stones [40]. Treatment of urolithiasis usually involves surgical removal of the stones. Unfortunately, it does not remove the causes of their formation [52]. Scientific research indicates a relationship between the occurrence of urolithiasis with diet, lifestyle, and climate [40,41,52].

## 7. Urolithiasis, Metabolic Acid, Inflammation, and Insulin Resistance

The mechanisms linking urolithiasis to the features of MS are not fully understood. Nevertheless, attention is often paid to the mild inflammation characteristic of MS, which also occurs in the presence of IR [53]. One of the most frequently studied inflammatory markers is interleukin-8 (IL-8/CXCL8). Its role in the development of neoplasms is often emphasised [54]. Its secretion is stimulated by hypoxia, reactive oxygen species, bacterial particles, and other cytokines such as IL-1, IL-6, or TNFα. The cells that secrete it are mainly leukocytes: blood monocytes, macrophages, epithelial and endothelial cells, and fibroblasts [54,55]. Suen et al. [56] focused on finding prognostic markers of urolithiasis and showed that the presence of urinary stones is associated with an inflammatory response, especially with increased levels of IL-8 in the urine. They suggest that IL-8 could be a marker as a screening test for urolithiasis. Wang et al. [57] showed that the values of pro-inflammatory cytokines, including IL-8 in plasma, decreased in patients after percutaneous nephrolithotripsy and suggested that IL-8 can be used as a predictive tool for patients after this procedure.

Another proposed explanation is the association of low ammonium excretion with urine in MS patients, which causes acidification of the urine [41,48]. Systemic metabolic acidosis alters the concentration of various substances in the urine and may contribute to stone formation. The occurrence of diet-induced metabolic acidosis activates kidney compensatory mechanisms to correct the acid-base balance. Removal of non-metallised anions or excretion of ammonium ions can then occur. This decreases urine pH, causing changes in its composition, hypocitraturia, hypercalciuria, and nitrogen and phosphorus loss. This predisposes to the formation of urinary stones [58]. Research by Kim et al. [59] showed that higher glucose levels and HOMA-IR index characterised men with nephrolithiasis. Such changes were not observed in the female population. Factors that predispose to insulin resistance are conditions characteristic of metabolic acidosis, such as insulin resistance in skeletal muscles and increased secretion of glucocorticosteroids and cortisol. Studies suggest that even minor deviations in pH towards metabolic acidosis reduce the sensitivity of tissues to insulin [58]. It can therefore be assumed that among patients with IR, an increased tendency to develop urolithiasis can be expected and vice versa, due to similar mechanisms causing these diseases, such as the presence of systemic metabolic acidosis, MS features, and the presence of inflammatory mediators such as interleukin 6 (IL-6) or tumor necrosis alpha (TNFα).

## 8. Diagnostics of IO

The gold standard in diagnosing insulin resistance is the hyperinsulinemic-euglycemic clamp (HEC). This method involves measuring the amount of glucose needed to maintain normal glucose levels constant during experimentally obtained hyperinsulinemia (corresponding to physiological postprandial concentrations). Unfortunately, it is not often used [60].

A simple diagnostic method used by doctors and nutritionists is to determine glucose and insulin levels at the beginning, after 1 h, and after 2 h of an oral glucose tolerance test (OGTT).

With these results, we can determine the risk of insulin resistance using the following guidelines:

- The reference value for fasting blood insulin levels should be between 3 and 17 mLU/L. Values above 17 mLU/L suggest the occurrence of insulin resistance [61].
- INS/GLU (Insulin/Glucose ratio) in 60 min of OGTT (mg/dL) should be less than 0.3. Values above 0.3 suggest the occurrence of insulin resistance [20].
- HOMA-IR (Homeostasis Model Assessment of Insulin Resistance) is calculated according to the formula based on fasting glucose and insulin measurement. A result above “1” suggests insulin resistance. Due to pulsatile insulin secretion, the measurement should be made 3 or 4 times and then the average of the measures should be treated as a result. Unfortunately, research is usually limited to one measurement [61,62,63,64,65]: -HOMA-IR = (GxI)/405 (when the glucose concentration is shown in mg/dL)-HOMA-IR = (GxI)/22.5 (when glucose is shown in mmol/L). The set of all formulas is presented in Table 2.

Specifying all parameters is extremely important because they can be mutually exclusive. This illustrates the results of Patient X in Table 3.

After analysing the data, we obtain contradictory results that are mutually exclusive; therefore, it is essential to perform extensive tests to determine the risk and occurrence of insulin resistance. Relying only on fasting insulin or HOMA-IR can give misleading results. For comparing patient X results with the reference values, see Table 4.

## 9. Interview and Anthropometric Measurements

It is crucial to conduct a proper dietary interview when we suspect insulin resistance. Elements worth paying particular attention to are:

- family medical history—especially the history of type 2 diabetes, hypertension, obesity, lipid disorders, polycystic ovary syndrome (PCOS)
- patient’s physical activity—the amount and type of activity performed, kind of work (sedentary, physical)
- dietary interview—the amount of simple and complex carbohydrates in the diet, the amount of alcohol consumed, the proportion of carbohydrates, proteins, and fats, the overall caloric content of the diet, covering the demand for microelements
- correct anthropometric measurements
- weight, waist circumference, WHR (waist-hip ratio) calculation, a visceral fat level
- environmental interview—determining the number of weight loss attempts, types of diets, eating habits.

## 10. Diet—Insulin Resistance and Acidosis

Patients with IR usually do not achieve the desired dieting therapy results after using a traditional low-calorie diet. In this case, it is necessary to introduce modifications to improve carbohydrate metabolism and reduce tissue resistance to insulin. The selection of the right amount and type of carbohydrates is crucial to lowering excess insulin production, which affects lipid metabolism processes in cells [62].

**Figure 4:**
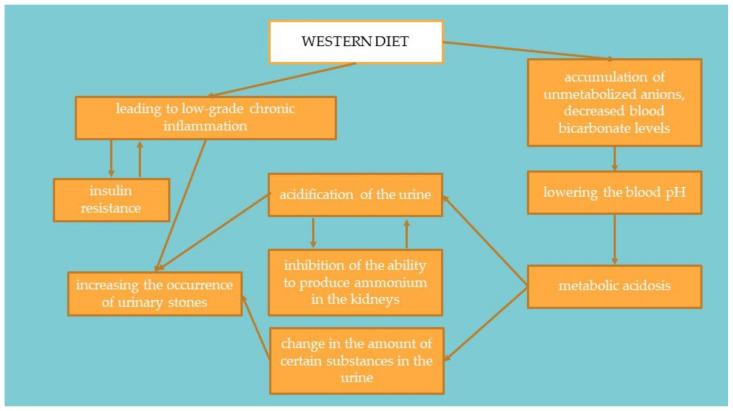
Linking the Western diet with insulin resistance and the occurrence of urolithiasis.

In countries with highly processed food consumption, urolithiasis is more common in the population, both among adults and children [40]. Foods rich in animal protein increase the production of metabolic acid compounds in the body, such as hydrogen chloride or bisulfate, which may affect the body’s acid-base balance [66,67,68,69,70]. When these foods are consumed, the acids are buffered by the lung excretion of CO_2_ and the production of sodium salts. These salts are then excreted through the kidneys, mainly with ammonium. Bicarbonate resulting from buffering is returned to the plasma as a substitute for bicarbonate used to soften the acids. When the production of acidic compounds exceeds their excretion via the lungs and kidneys, the amount of bicarbonate in the plasma decreases and thus the blood pH decreases [70]. This mechanism is presented in Figure 4. Studies suggest that treatment of metabolic acidosis with bicarbonate increases insulin sensitivity; therefore, to correct the acid-base balance, it is recommended to significantly increase the amount of alkaline-forming vegetables and fruits in the diet [69,71]. It has been proven that people on a plant-based diet have substantially lower HOMA-IR values and blood glucose levels. It suggests that a diet rich in plant products and limiting animal foods may significantly affect the development of IR [72].

### 10.1. Carbohydrates

Scientific reports regarding the recommendations for the number of carbohydrates in the diet seem contradictory. They suggest that both a high-carbohydrate diet and a low-carbohydrate diet can benefit patients. Therefore, the number of carbohydrates in dietary therapy should be individually adjusted for each patient based on its effects. Too low an amount of carbohydrates can adversely affect the brain and initiate ketone bodies’ production; too much causes excessive insulin secretion and, consequently, no diet effects. Regardless of the chosen dietary direction, most carbohydrates consumed by the patient should be high-fibre vegetables and then grain products with a low glycemic index. The remaining amount should be filled with fruit and a low index. Berries are especially recommended, such as strawberries, raspberries, and blueberries. It should be emphasised to significantly reduce alcohol consumption, increasing blood glucose [65,73,74,75]. In addition, attention should be paid to the excessive use of sweeteners, which can significantly increase carbohydrate-induced insulin secretion [15].

### 10.2. Proteins

**Figure 5:**
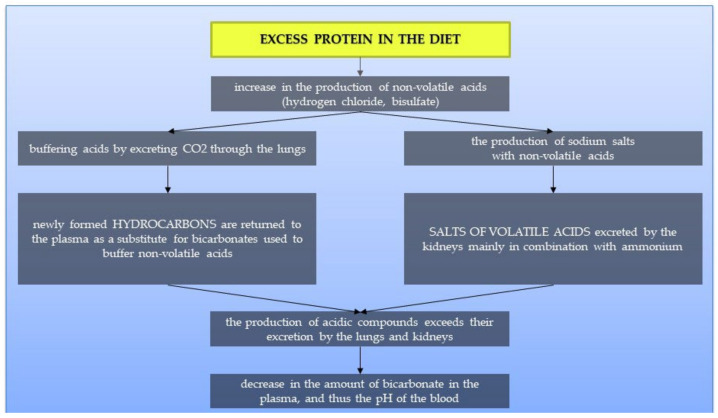
Effect of a high-protein diet on blood pH.

If it seems justified to reduce the number of carbohydrates in the patient, then the amount of protein should be increased (about 15–20%). However, it should be remembered that too much animal protein causes acidification. Therefore, an appropriate amount of alkaline-forming vegetables and fruits should be selected to reduce the possibility of acidification of the organism [69,71]. The diet’s primary protein sources should be lean meat, unsweetened dairy products without additives (semi-fat products are recommended), legumes, and fatty marine fish containing large amounts of Omega-3 fatty acids. In lipid disorders, it is necessary to reduce red meat significantly. The addition of products including protein to meals with carbohydrates improves the body’s sensitivity to insulin; therefore, it is worth combining dishes, e.g., fruit and kefir cocktails or as additives sauces based on cream or yoghurt for carbohydrate dishes [76,77]. The mechanism of the influence of a high-protein diet on blood pH is presented in Figure 5.

### 10.3. Fats

Fats delay gastric emptying and absorption of nutrients, including glucose. Thanks to this, they prolong their metabolism. However, it should be pointed out that IR is usually accompanied by overweight or obesity, so the quality of fat taken by the patient is extremely important. Scientific reports indicate that the composition of fatty acids significantly influences IO’s development due to their role in the correct arrangement of cell membrane lipids. Maintaining an adequate omega-three and omega-six fatty acids ratio in the diet is recommended. Cold-pressed unrefined oils are best suited, for example, rapeseed oil or olive oil. We should avoid products rich in saturated fatty acids, such as fatty meat, lard, and palm oil [11,78].

## 11. Tips for Laying Diets

To properly balance the patient’s diet, follow the rules usually used during composing diets for people with diabetes. We can include, among others:

- eliminating juices and sweetened drinks, replacing them with water and tea (herbal, green, red)
- avoiding fruit in processed form, for example, juices, nectars, mousses, jams, because the sugar they contain is absorbed much faster than in the case of unprocessed fruits
- properly composed meals—combine products with a higher amount of carbohydrates with products containing fat and protein—this will slow down the absorption of glucose into the blood
- proper planning of meal times—eating regularly and at regular intervals.

Moreover, it is worth paying attention to the type of food when shopping. Common mistakes made by patients include:

- choosing highly processed bread, e.g., rice cakes or crispbread, buying bread containing caramel, malt, or honey
- use of products containing large amounts of salt and preservatives, e.g., broth cubes, spice mixtures, flavour enhancers, Maggi
- buying light products with low-fat content is often offset by an additional portion of sugar in the product.

These errors often result from the lack of proper nutrition education, which should occur during the first visit to the dietitian.

## 12. Physical Activity

Physical activity is a critical element of therapy for insulin resistance. During increased physical activity, when the amount of oxygen supplied to the muscles is too low, the cells begin to conduct anaerobic glucose metabolism. This process results in a decrease in ATP in the cell and a simultaneous increase in 5’AMP-activated protein kinase (AMPK), which regulates the body’s homeostasis. Consequently, there is an increase in GLUT 4 (Glucose transporter type 4) in the cell membrane and increased glucose uptake by cells. Moreover, AMPK causes (a) inhibition of cholesterol, (b) TAG synthesis, (c) oxidation of fatty acids in the liver and muscles, and (d) inhibition of lipolysis and (5) lipogenesis in adipocytes. It has been proven that the risk of IR among active people is lower by 33–50% compared to people who do not participate in sports [79,80,81].

## 13. Insulin Resistance and Urolithiasis in the Literature

In recent years, many studies focusing on IR have appeared. Cummings et al. proved that dietary intervention in overweight children to reduce the consumption of sweet drinks has a significant impact on the HOMA index’s value [82]. Buscemi et al. [83] tested the blood of over 500 people and showed that a Mediterranean diet significantly improves insulin sensitivity. Scientists create detailed recommendations for the right amount of carbohydrates in the diet. Due et al. [65] confirmed in their research thesis that a diet rich in monounsaturated fatty acids has a better effect on fasting glucose and insulin than a low-fat diet. O’Neill [75] came to similar conclusions, proposing therapeutic carbohydrate restriction based on his research results. These arguments are opposed by the research of Kahleov et al. [73], in which it turned out that a plant-based high-carbohydrate diet was also associated with improved insulin resistance. In the literature, the topic of the association of IR with urolithiasis is not widespread; however, researchers are suggesting the association of urolithiasis with MS, of which IR is a component. Johnson et al. [84] studied over 30 people taking fructose every day and assessed their uric acid levels. It has been proven that the increased consumption of fructose was associated with an increase in the urinary content and a decrease in blood pH, which is a characteristic feature of MS and IR. Interesting observations about the relationship of adiponectin with diabetes, insulin, and urolithiasis were presented by Devasia et al. [85]. This may indicate that the increase in adiponectin compensates further development of these diseases by addressing insulin sensitivity. More research is needed to explore these mechanisms [85].

## 14. Conclusions

Insulin resistance impacts the development of many diseases, such as type 2 diabetes, hypertension, and lipid profile disorders. It is also associated with urolithiasis. Although its exact mechanism has not yet been fully understood, there are dietary guidelines that we can follow during patient therapy. The most important of them include selecting the quantity and quality of products containing complex carbohydrates, avoiding simple carbohydrates, choosing healthy sources of fat, and proper composition and preparation of meals. Physical activity supporting the body’s metabolic changes is also critical. Using these guidelines can adequately help a patient’s therapy with insulin resistance and urolithiasis and delay or inhibit the occurrence of complications and other diseases. However, we should still pay special attention to these diseases studies because they can provide tips to improve nutritional treatment.

## Figures and Tables

**Table 1:** Criteria for the diagnosis of metabolic syndrome based on the consensus of the International Diabetes Federation (IDF) and the American Heart Association/National Heart, Lung, and Blood Institute (AHA/NHLBI) from 2009 [44].

Identification of at Least 3 of the Listed Factors	
Component	Value
Incorrect waist size	- Caucasian, Middle East, and Mediterranean population: 94 cm (M), 80 cm (F) - USA population: 102 cm (M), 88 cm (F) - Asian population: 90 cm (M), 80 cm (F)
Triglycerides	150 mg/dL or appropriate therapy
HDL cholesterol	40 mg/dL (M), 50 mg/dL (F), or appropriate therapy
Blood pressure	130/85 mmHg or appropriate therapy
Fasting blood glucose	100 mg/dL or using appropriate therapy

M—male, F—female.

**Table 2:** Determination of parameters and the occurrence of insulin resistance based on insulin and glucose measurement after OGTT administration [20,61,62,63,64,65].

Parameter	Reference Value
The concentration of insulin in the fasted blood	3–17 mLU/L
Insulin/Glucose ratio (mg/dL)	insulin resistance when >0.3
HOMA-IR = (fasting glucose × fasting insulin)/405 (when the glucose concentration is given in mg/dL)	insulin resistance when >1
HOMA-IR = (fasting glucose × fasting insulin)/22.5 (when glucose is given in mmol/L)	insulin resistance when >1

**Table 3:** Patient X serum concentrations of selected parameters.

Parameter	Result [mg/dL]
fasting glucose	89
glucose after 1 h	176
glucose after 2 h	136
fasting insulin	26.5
insulin after 1 h	165.3
insulin after 2 h	199.5

**Table 4:** Comparing patient X results with reference values.

Parameter	Reference Value/Standard	Patient’s X Result
The concentration of insulin in the fasted blood	3–17 mLU/L	26.5—incorrect
Insulin/Glucose ratio (mg/dL)	insulin resistance when >0.3	0.29—correct
HOMA-IR	insulin resistance when >1	5.8—incorrect

## Data Availability

All necessary data are included in the paper.
